# Inhibition mechanism of naphthylphenylamine derivatives acting on the CDC25B dual phosphatase and analysis of the molecular processes involved in the high cytotoxicity exerted by one selected derivative in melanoma cells

**DOI:** 10.1080/14756366.2020.1819257

**Published:** 2020-09-29

**Authors:** Federica Aliotta, Rosarita Nasso, Rosario Rullo, Alessandro Arcucci, Angelica Avagliano, Martina Simonetti, Gennaro Sanità, Mariorosario Masullo, Antonio Lavecchia, Maria Rosaria Ruocco, Emmanuele De Vendittis

**Affiliations:** a Department of Molecular Medicine and Medical Biotechnology, University of Naples Federico II, Naples, Italy; b Department of Movement Sciences and Wellness, University of Naples “Parthenope”, Naples, Italy; c Institute for the Animal Production Systems in the Mediterranean Environment, CNR, Naples, Italy; d Department of Public Health, University of Naples Federico II, Naples, Italy; e Department of Pharmacy, “Drug Discovery” Laboratory, University of Naples Federico II, Naples, Italy

**Keywords:** CDC25, inhibition mechanism, melanoma, cytotoxicity, anticancer agents

## Abstract

The dual phosphatases CDC25 are involved in cell cycle regulation and overexpressed in many tumours, including melanoma. CDC25 is a promising target for discovering anticancer drugs, and several studies focussed on characterisation of quinonoid CDC25 inhibitors, frequently causing undesired side toxic effects. Previous work described an optimisation of the inhibition properties by naphthylphenylamine (NPA) derivatives of NSC28620, a nonquinonoid CDC25 inhibitor. Now, the CDC25B•inhibitor interaction was investigated through fluorescence studies, shedding light on the different inhibition mechanism exerted by NPA derivatives. Among the molecular processes, mediating the specific and high cytotoxicity of one NPA derivative in melanoma cells, we observed decrease of phosphoAkt, increase of p53, reduction of CDC25 forms, cytochrome *c* cytosolic translocation and increase of caspase activity, that lead to the activation of an apoptotic programme. A basic knowledge on CDC25 inhibitors is relevant for discovering potent bioactive molecules, to be used as anticancer agents against the highly aggressive melanoma.

## Graphical Abstract

**Figure:**
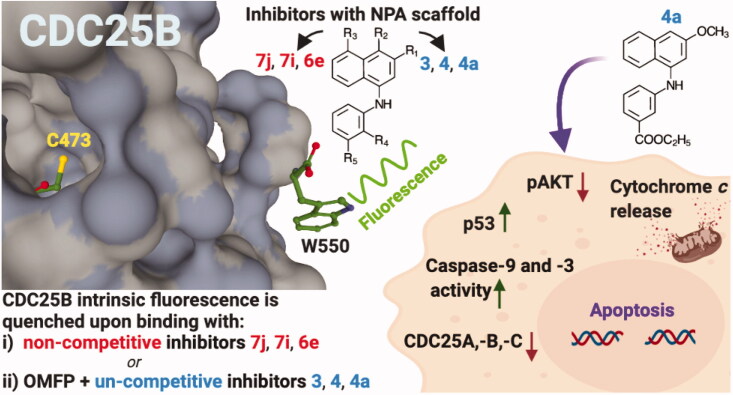

## 1. Introduction

Although the different types of cancer are characterised by a great heterogeneity, one of the main and more common traits is represented by a deregulation of the cell cycle. Indeed, many (chemo)therapeutic agents act on cell cycle through the inhibition of macromolecules involved in the regulation of cell cycle progression^1^. Cyclin-dependent kinases (CDKs) are pivotal regulators of cell cycle; they are active when linked to specific cyclins and their regulation involves the binding to negative modulators and/or the occurrence of phosphorylation/dephosphorylation events^1^^,^^2^. In particular, the CDK•cyclin complexes are inactivated through the phosphorylation of specific residues of threonine and tyrosine in the CDK subunit. The control of cell cycle progression also involves the action of CDC25 enzymes, catalysing the removal of the inhibitory phosphate groups in CDKs^3^. Indeed, the dephosphorylation of CDK threonine and tyrosine residues is catalysed by CDC25 dual phosphatases, highly conserved and present in all eukaryotes, except plants^4–6^. There are three forms of CDC25 enzymes, CDC25A, –B and –C. CDC25A is mainly involved in the regulation of both G1/S and G2/M transitions. In particular, in G1 phase CDC25A dephosphorylates and thus activates CDK4 and CDK6, and then, in late G1 phase promotes the G1/S transition, by dephosphorylating and activating the CDK2•cyclin A and CDK2•cyclin E complexes^5^. Furthermore, CDC25A also activates the CDK1•cyclin B complex, thus contributing to the G2/M progression^7^. The CDC25B-mediated dephosphorylation of Thr14 and Tyr15 residues in CDK1 activates this kinase and promotes the cell entry into mitosis. Thus, CDC25B can be considered as a mitotic inducer, because it participates in the cytoplasmic CDK1•cyclin B activation. The main role of CDC25C is to maintain the activated state of the CDK1•cyclin B complex in the nucleus, thus ensuring a full progression into mitosis^4^^,^^8^^,^. However, in adult tissues, the functions of three CDC25 paralogues can be compensatory among them^9^^,^^10^^,^.

**Figure 1.**
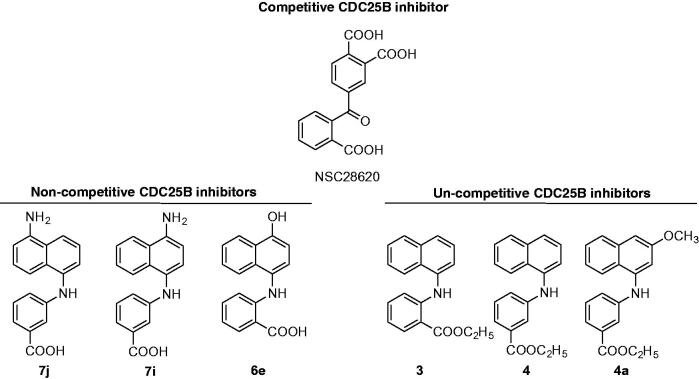
Chemical structure of CDC25B inhibitor NSC28620 and selected NPA derivatives.

An overexpression of CDC25, mainly –A and –B enzymes, has been observed in many cancers, including melanoma, a finding frequently associated to tumour increased aggressiveness and fatal prognosis^11–14^. Hence, CDC25 phosphatases are considered potential targets for anti-neoplastic therapies. To date, different types of molecules endowed with CDC25 inhibitory action have been described. Some of them exert a toxic effect in tumour cells^15–18^, whereas others induce a decrease of tumour size through its growth arrest, when tested on mice-xenografted human tumours^19–21^. CDC25 inhibitors can be classified as quinonoids, thiophenic derivatives, electrophilic inhibitors, phosphate surrogates or coumarins^22–29^. However, none of CDC25 inhibitors have been further selected for *in vivo* experiments, because of their low activity and poor selectivity^8^. Hence, the synthesis and characterisation of new molecules endowed with an increased inhibitory activity towards CDC25 and lower adverse effects are required. In a previous work, we have reported the biochemical and biological characterisation of NSC28620 (Figure 1), a nonquinonoid reversible competitive inhibitor of CDC25B endowed with *K*_i_ 5.3 µM, that reduced the cell growth rate of some tumour cells, when added at 200 µM^16^. An optimisation programme on this lead compound was realised, and 31 derivatives with naphthylphenylketone (NPK) and naphthylphenylamine (NPA) structure were obtained^18^. Some of the NPA derivatives displayed a higher inhibition power compared to the lead compound, such as the most active one, **7j** (Figure 1), having a *K*_i_ value of 0.8 µM. Contrary to NSC28620, derivatives were apparently characterised by two main types of reversible inhibition, that is, noncompetitive or uncompetitive. Furthermore, one NPA derivative, **4a**, when added at 10 µM, significantly reduced the cell growth of melanoma cells through an arrest in the G2/M phase of cell cycle and increased the phosphoCDK1 (pCDK1) protein levels.

In this work, we have performed a deeper investigation on the interaction between CDC25B and selected NPA derivatives of the lead NSC28620: compounds **7j**, **7i** and **6e** as representatives of putative noncompetitive inhibition, and compounds **3**, **4** and **4a** as representatives of putative uncompetitive inhibition (Figure 1).

The previous study showed that the interaction between CDC25B and its inhibitors could be followed through measurements of intrinsic fluorescence of the purified recombinant enzyme, thanks to the presence of a single tryptophan (W550) in its catalytic domain^18^. A deeper insight on this powerful tool was realised by using wild-type and mutant C473S version of CDC25B, thus allowing the measurement of parameters related to direct or substrate-mediated interaction between the enzyme and its ligands. Furthermore, the previous work demonstrated that, among the NPK and NPA derivatives, only compound **4a** exerted a significant antiproliferative action at a low dose in melanoma cells^18^. Here, we have better characterised the mechanism mediating the toxic action of **4a** in melanoma cells. In particular, we have shown that this inhibitor induces the activation of an apoptotic programme, mainly mitochondria- and caspase-mediated. In addition, **4a** reduces the protein levels of all three forms of CDC25 and affects the expression of typical proliferation and apoptotic markers, such as phosphoAkt (pAkt) and p53.

## 2. Materials and methods

### 2.1. Materials and reagents

Stock solutions of the NPA derivatives of NSC28620 were prepared in dimethylsulphoxide (DMSO) at 20 mM concentration; when diluted in the aqueous solution, the final concentration of the various compounds never exceeded 50 µM. The stock solution of O-methylfluorescein phosphate (OMFP) was prepared in methanol at 2 mM concentration. For the production of the recombinant form of CDC25B catalytic domain, a heterologous expression system was used, constituted by the vector pET28a-CDC25B-cd, kindly provided by H. Bhattacharjee (Florida International University, Herbert Wertheim College of Medicine, Miami, Florida) and the *Escherichia coli* BL21(DE3) strain from Novagen (Madison, WI, USA); the purification procedure was essentially as previously described^18^. Dulbecco’s modified Eagle’s medium (DMEM), Medium 199, foetal bovine serum (FBS), L-glutamine, penicillin G, streptomycin, and trypsin were bought from Lonza (Milano, Italy). Isopropyl-β-thiogalactopyranoside (IPTG), Tris-(2-carboxyethyl)-phosphate hydrochloride (TCEP), OMFP, propidium iodide (PI) were purchased from Sigma Aldrich (St. Louis, MO, USA). A protease inhibitor cocktail was obtained from Roche Diagnostics (Indianapolis, IN, USA). Caspase-3 and caspase-9 fluorimetric assay kits were purchased from BioVision (Milpitas, CA, USA). The pancaspase inhibitor Z-VAD-FMK was purchased from Selleckchem (Houston, TX, USA). Rabbit monoclonal antibody against GAPDH was purchased from Cell Signalling Technology (Danvers, MA, USA). Mouse monoclonal antibody against COX-IV was purchased from Elabscience (Houston, TX, USA). Mouse monoclonal antibody against β-tubulin was purchased from Sigma-Aldrich. FITC donkey, anti-mouse secondary antibody was purchased from Jakson ImmunoResearch (Suffolk, UK). Rabbit polyclonal antibodies against CDC25B, pAkt (Ser473) or Akt and mouse monoclonal antibodies against cytochrome *c*, CDC25A, CDC25C or p53, as well as HRP conjugated secondary antibody were purchased from Santa Cruz Biotechnology (Heidelberg, Germany). All other reagents and solvents of high analytical grade were from Sigma-Aldrich.

### 2.2. Preparation of CDC25B-C473S

The mutant form of CDC25B, carrying the C473S replacement (CDC25B-C473S), was obtained through site-directed mutagenesis of the CDC25B catalytic segment cloned in pET28a-CDC25B-cd. To this aim, the QuikChange site-directed mutagenesis kit (Stratagene, La Jolla, CA, USA) and the forward and reverse primers for C473S replacement (5′d-ATCCTCATTTTCCACT*C*TGAATTCTCATCTGAG-3′ and 5′d-CTCAGATGAGAATTCA*G*AGTGGAAAATGAGGAT-3′) were used. Positive clones were obtained, and the nucleotide sequencing of the mutagenised segment excluded the presence of undesired mutations. The new vector was called pET28a-CDC25B-C473S-cd. The expression of the new vector and the purification of the recombinant form of CDC25B-C473S catalytic domain were carried out as previously reported for wild-type CDC25B catalytic domain^18^, and similar yields were obtained. As expected, the mutant enzyme was unable to hydrolyse the synthetic substrate OMFP, when its activity was checked with the fluorimetric assay method previously described^18^.

### 2.3. Fluorescence studies on the purified catalytic domain of CDC25B and CDC25B-C473S

Fluorescence spectra were recorded at 20 °C using a computer-assisted Cary Eclipse spectrofluorimeter (Varian, Palo Alto, CA, USA) equipped with an electronic temperature controller. The excitation wavelength was set at 280 nm and the excitation and emission slits were set at 10 and 20 nm, respectively. A 500 µL final volume of a 0.2 µM solution of CDC25B or CDC25B-C473S dissolved in 20 mM Tris•HCl, pH 7.8, 0.5 mM TCEP was directly prepared in the fluorimetric cuvette in the absence or in the presence of OMFP and/or some NPA derivatives. Serial pre-dilutions of the NPA derivatives or OMFP at increasing concentration were prepared in DMSO or methanol, respectively. Alongside, a convenient amount of the enzyme pre-dilution was prepared just before its usage for the experiment and stored on ice. When the combined effect of NPA derivative and OMFP was investigated, two enzyme pre-dilutions were prepared in the absence or in the presence of 5 µM OMFP. In order to evaluate the specific effect produced by any addition of solvent/ligand on the intrinsic fluorescence of the enzyme, 495 µL of the enzyme pre-dilution were placed in the fluorimetric cuvette and the relative spectrum was recorded; after the addition of 5 µL of each pre-dilution of solvent/ligand and fast mixing, the spectrum was recorded again. This procedure, repeated for each experimental point, allowed a normalisation of the specific effects produced by solvent/ligands. Furthermore, a correction was also applied for the little inner-filter effect due to the absorbance at 280 nm of the NPA derivative used at different concentrations; on the other hand, no correction was necessary for OMFP. All spectra, recorded at a scan speed of 120 nm/min, were normalised and corrected.

### 2.4. Cell cultures

The human melanoma cell lines A375 and A2058, deriving from a primary tumour or lymph nodal metastasis, respectively, were kindly provided from CEINGE (Naples, Italy). Cells were grown in DMEM supplemented with 10% FBS, 2 mM L-glutamine, 100 IU/mL penicillin G and 100 µg/mL streptomycin in humidified incubator at 37 °C under 5% CO_2_ atmosphere. The cell line BJ-5ta, a human skin fibroblast immortalised with the human telomerase reverse transcriptase^30^ was cultured in a 4:1 mixture of DMEM and Medium 199 supplemented with 4 mM L-glutamine, 4.5 g/L glucose, 1.5 g/L sodium bicarbonate, 10% FBS, 100 IU/mL penicillin G, 100 mg/mL streptomycin in humidified incubator at 37 °C under 5% CO_2_ atmosphere. All cells were split and seeded every 3 days and used during their exponential phase of growth. Cell treatments were usually carried out after 24 h from plating.

### 2.5. Evaluation of apoptosis

Cytofluorimetric analysis was used to determine the number of apoptotic nuclei essentially as previously described^31^. Briefly, cells were seeded into six-well plates at 3 × 10^5^ cells/well and after 24 h were treated with **4a** or 0.5% (v/v) DMSO and incubated for different times. Then, cells were harvested with trypsin, and centrifuged, and the pellets were resuspended in 200 µL of a hypotonic lysis solution containing 50 µg/mL PI. After incubation at 4 °C for 30 min, cells were analysed with a FACScan flow cytometer (Becton Dickinson, Franklin Lakes, NJ, USA) to evaluate the presence of nuclei with DNA content lower than diploid.

### 2.6. Measurement of caspase-3 and caspase-9 activity

The caspase-3 and caspase-9 enzymatic activity was measured by using caspase-3 and -9 fluorimetric assay kits, respectively, according to the manufacturer’s protocol. Briefly, cells were seeded into 75 cm^2^ plates (2 × 10^6^ cells/plate) and after 24 h treated with **4a** or 0.5% (v/v) DMSO for 24 h. At the end of incubation, cells were collected, washed with phosphate-buffered saline constituted by 10 mM Na_2_HPO_4_, 2 mM KH_2_PO_4_, pH 7.4, supplemented with 137 mM NaCl and 2.7 mM KCl (PBS), and finally lysed at 4 °C in the cell lysis buffer. Cell lysates were incubated with 50 µM DEVD-AFC or LEHD-AFC substrates at 37 °C for 2 h, to detect caspase-3 or caspase-9 activity, respectively, using a Cary Eclipse spectrofluorimeter. Excitation and emission wavelengths were set at 400 nm and 505 nm, respectively; both excitation and emission slits were set at 10 nm.

### 2.7. Total cell lysates and subcellular fractionation for Western blotting analysis

To obtain the total protein extract, cells were seeded into 6-well-plates (3 × 10^5^ cells/plate) for 24 h at 37 °C and then treated with **4a** or 0.5% (v/v) DMSO. After treatment, cells were harvested, washed with PBS, and then lysed in ice-cold modified radio immunoprecipitation assay (RIPA) buffer (50 mM Tris•HCl, pH 7.4, 150 mM NaCl, 1% Nonidet P-40, 0.25% sodium deoxycholate, 1 mM Na_3_VO_4_ and 1 mM NaF), supplemented with protease inhibitors and incubated for 30 min on ice. The supernatant, obtained after centrifugation at 13200 *g* for 30 min at 4 °C, constituted the total protein extract. Protein concentration was determined by the Bradford method, using bovine serum albumin (BSA) as standard^32^. To obtain the cytosolic and mitochondrial fractions, cells were plated at a density of 2 × 10^6^ cells/plate for 24 h at 37 °C. After the treatment with **4a** or 0.5% (v/v) DMSO, cells were harvested, washed in PBS, and then resuspended in buffer A (5 mM Hepes, pH 7.4, 250 mM mannitol, 0.5 mM EGTA, 0.1% BSA), supplemented with protease inhibitors, and homogenised. The homogenate was centrifuged at 600 *g* for 5 min at 4 °C and the supernatant was then centrifuged at 10300 *g* for 10 min at 4 °C. The resulting supernatant represented the cytosolic fraction, whereas the pellet, constituting the mitochondrial fraction was resuspended in RIPA buffer. Protein concentration was determined as previously indicated. Equal amounts of total, cytosolic or mitocondrial protein extracts were used for Western blotting analysis. Briefly, protein samples were dissolved in SDS-reducing loading buffer, run on sodium dodecylsulfate polyacrylamide gel electrophoresis (SDS/PAGE), and then transferred to Immobilon P membrane (Millipore, Saint Louis, MO, USA). The filter was incubated with the specific primary antibody at 4 °C overnight and then with the secondary antibody at room temperature for 1 h. Membranes were then analysed by an enhanced chemiluminescence reaction using WesternBright ECL (Advansta, San Jose, CA, USA) according to manufacturer’s instructions; signals were visualised by autoradiography. The analysis of pAkt protein levels was performed as previously described^33^. Briefly, filters were first incubated with the anti-pAkt primary antibody and then with the secondary antibody and membranes were analysed by an enhanced chemiluminescence reaction as previously indicated. The same filters were then stripped, using AbCam (Cambridge, UK) stripping solution, pH 2 (200 mM glycine, 3.5 mM SDS, 1% Tween-20) at 45 °C for 30 min, washed 3 times with PBS-Tween 0.1%, incubated with anti-Akt primary antibody and then with the relative secondary antibody. Signals were detected as previously specified.

### 2.8. Immunofluorescence staining

Immunofluorescence experiments were carried out essentially as previously described^34^. Briefly, cells were plated on glass coverslips at a density of 3 × 10^5^ cells/well in 6-well plates, and after 24 h were treated with **4a** or 0.5% (v/v) DMSO. At the end of treatment, cells were incubated with 90 nM MitoTracker Red (Invitrogen, Inchinnan, UK) at 37 °C for 1 h and then washed three times with ice-cold PBS. Cells were fixed with 4% paraformaldehyde, and then blocked in donkey serum (Millipore) diluted 1:10 in PBS, for 30 min at room temperature. Glass coverslips were incubated for 1 h at 37 °C with a mouse monoclonal antibody against cytochrome *c*, diluted 1:50 in PBS, washed three times with PBS and subsequently incubated for 1 h at 37 °C with a FITC donkey anti-mouse secondary antibody diluted 1:50 in PBS. After three washings in PBS, the cell nuclei were labelled with DAPI (Vector Laboratories, Inc., Burlingame, CA, USA). Glass coverslips mounting was done in Vectashield (Vector Laboratories). The images were taken with digital camera connected to the microscope (Leica DFC345FX, Leica Microsystems, Wetzlar, Germany) and then merged with the software Leica Application Suite 3.6.

### 2.9. Statistical analysis

Data are reported as the mean ± standard error (SE). The statistical significance of differences among groups was evaluated using ANOVA, with the Bonferroni correction as *post hoc* test or the Student’s *t*-test where appropriate. The significance was accepted at the level of *p* < 0.05.

## 3. Results and discussion

### 3.1. Intrinsic fluorescence of CDC25B and its C473S mutant

The presence of a single tryptophan residue, W550, in the catalytic domain of CDC25B is particularly attracting for analysing the binding interaction with its ligands through a spectrofluorimetric analysis. Indeed, W550 is located on the edge of the CDC25B region called swimming pool and has a great exposure to the water environment, as suggested by the position of its emission maximum in the fluorescence spectrum^18^. Furthermore, the swimming pool is adjacent to the catalytic site of CDC25B, and several ligands of this enzyme have been proposed to bind directly into this concave pocket or into a larger region including both swimming pool and catalytic site^24^^,^^25^^,^^35^.

Among the various ligands of CDC25B, the OMFP represents the common synthetic substrate used to measure the phosphatase activity of all CDC25 forms. Upon its binding to the enzyme, the nonfluorescent OMFP is hydrolysed to the fluorescent product OMF, thus allowing a measurement of the catalytic activity through the rate of OMF formation. In order to study the interaction between CDC25B and OMFP without the “complication” of its hydrolysis, a recombinant mutant CDC25B with the catalytic C473 residue replaced by serine was designed and prepared (CDC5B-C473S). As expected, the mutant enzyme is unable to hydrolyse OMFP (not shown), but has an emission spectrum almost superimposable to that of wild-type CDC25B, with only a minimum red-shift of its maximum (Supplementary Figure 1). In order to analyse the effects of various ligands on the emission spectrum of both wild-type and C473 mutant CDC25B, we have chosen 357 nm as a common wavelength for the emission maximum. These observations indicate that the C473S replacement renders the enzyme inactive, but does not essentially alter the fluorescent emission of the enzyme. Therefore, we took advantage of these properties to study the interaction between the enzyme and substrate in the absence of catalysis.

### 3.2. Interaction between CDC25B-C473S and the substrate OMFP evaluated through intrinsic fluorescence measurements

**Figure 2.**
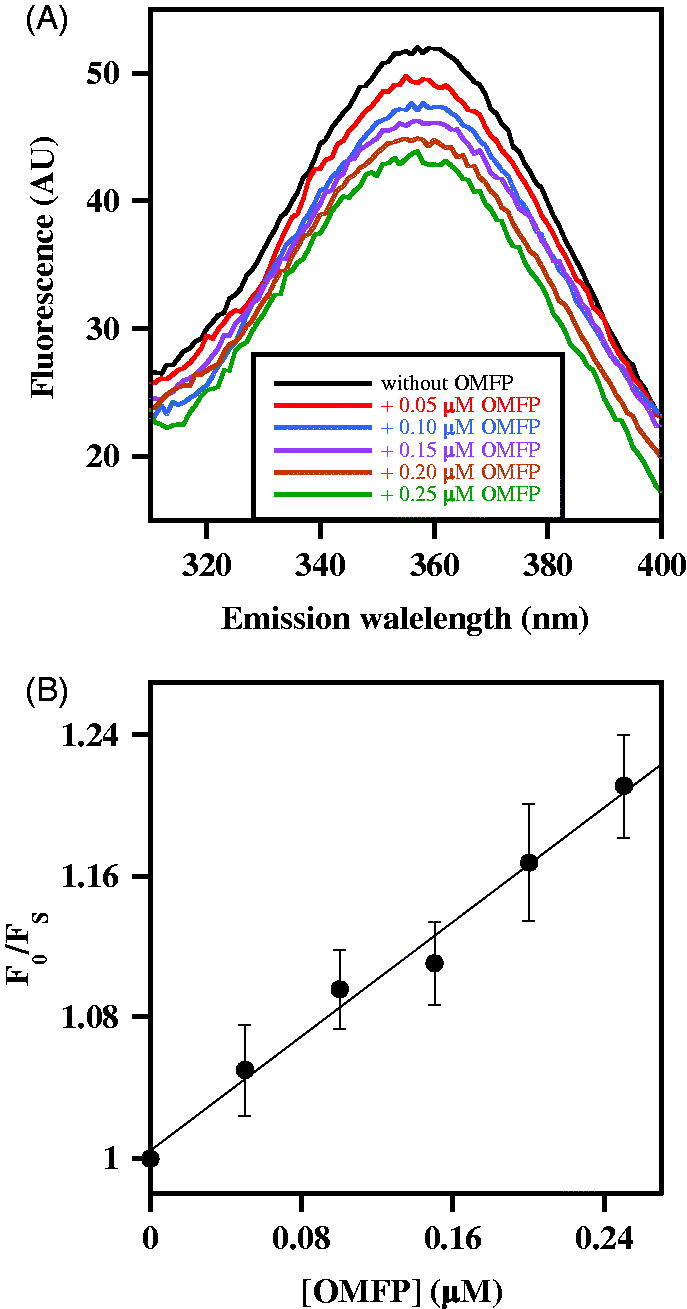
Effect of OMFP on the intrinsic fluorescence of recombinant CDC25B-C473S. (a) The fluorescence spectra of 0.2 µM CDC25B-C473S in the absence or in the presence of the indicated OMFP concentration were recorded and normalised as indicated in Materials and Methods. An identical 1% (v/v) final concentration of both methanol and DMSO is present in all samples. (b) The data of fluorescence emission at 357 nm obtained in the absence (F_0_) or in the presence of each OMFP concentration (F_S_) were treated according to the Stern–Volmer equation.

The effects produced by an increasing OMFP concentration on CDC25B-C473S fluorescence spectrum were evaluated. In the presence of OMFP, a dose-dependent quenching of the fluorescence yield is observed, without a shift of the emission maximum (Figure 2(a)). This behaviour allows an evaluation of the parameters governing the enzyme•ligand interaction, by comparing the fluorescence emission at 357 nm obtained at each substrate concentration (F_S_) with the corresponding value obtained in the absence of substrate (F_0_). The data were then analysed with the Stern-Volmer equation, F_0_/F_S_ = 1 + *K*_SV_ [Q], stating that the fluorescence reduction (F_0_/F_S_) of the fluorophore linearly increases with the quencher (Q) concentration. Indeed, the data fit a straight line starting from F_0_/F_S_ = 1 with a slope equal to the Stern-Volmer constant *K*_SV_ (Figure 2(b)). This result suggests that the interaction between CDC25B-C473S and OMFP causes a dose-dependent quenching of the intrinsic fluorescence due to W550. The value of *K*_SV_ extrapolated from the equation is 0.814 µM^−1^ and its reciprocal represents the OMFP concentration, i.e. 1.23 µM, leading to 50% quenching of CDC25B-C473S fluorescence emission. This value could also correspond to the equilibrium dissociation constant (*K*′_D_) of the enzyme•substrate complex. Interestingly, this value is even lower than the affinity constant *K*_M_ of the wild type CDC25B for the substrate OMFP, as previously reported (*K*_M_ = 2.7 ± 0.2 µM)^18^. Therefore, these data suggest that CDC25B-C473S tightly binds the substrate in a way similar to that of CDC25B.

### 3.3. Interaction between CDC25B and its inhibitors evaluated through intrinsic fluorescence measurements

**Figure 3.**
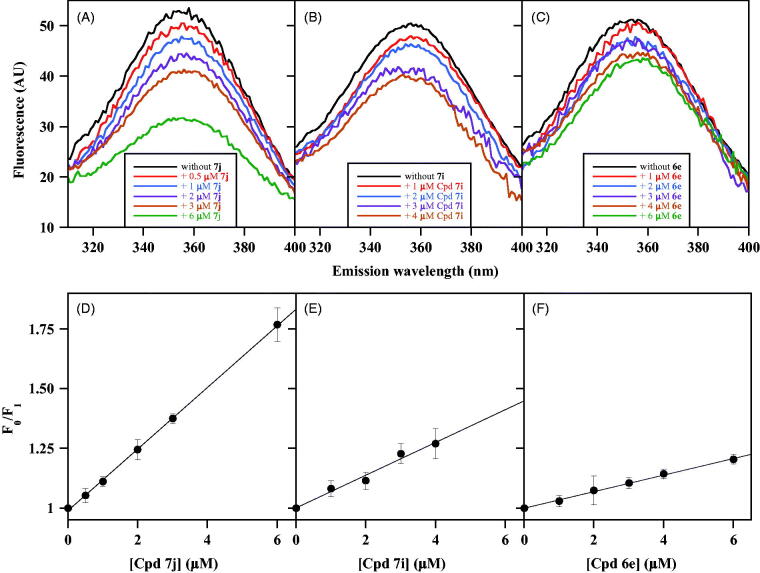
Effect of some noncompetitive inhibitors on the intrinsic fluorescence of recombinant CDC25B. The fluorescence spectra of 0.2 µM CDC25B in the absence or in the presence of the indicated concentrations of **7j** (a), **7i** (b) or **6e** (c) were recorded, normalised and corrected as indicated in Materials and Methods. An identical 1% (v/v) final concentration of DMSO is present in all samples. The data of fluorescence emission at 357 nm obtained in the absence (F_0_) or in the presence of each inhibitor concentration (F_I_) for **7j** (d), **7i** (e) or **6e** (f) were treated according to the Stern–Volmer equation.

The intrinsic fluorescence of CDC25B represents a powerful tool for investigating the interaction of the enzyme with its inhibitors. According to the mechanism of inhibition investigated by Lineweaver–Burk plots of CDC25B phosphatase activity, the various NPA derivatives of NSC28620 have been putatively assigned to two main groups, that is, noncompetitive or uncompetitive inhibitors. In particular, the compounds chosen as representative members of noncompetitive inhibition are **7j**, **7i** and **6e** having *K*_i_ of 0.8 µM, 1.4 µM and 2.7 µM, respectively. Figure 3 reports the effects produced by these noncompetitive inhibitors on the intrinsic fluorescence of CDC25B. The emission spectra obtained in the presence of an increasing concentration of **7j** (Figure 3(a)), **7i** (Figure 3(b)) or **6e** (Figure 3(c)) indicate that each inhibitor causes an evident dose-dependent quenching of the fluorescence signal, although with different effects probably depending on the relative inhibition power. Also in this case, no shift of the emission maximum is observed, and therefore, the fluorescence signal at 357 nm at each inhibitor concentration (F_I_) is compared with the corresponding value obtained in the absence of inhibitor (F_0_), using the Stern–Volmer equation. From the corresponding plots of **7j** (Figure 3(d)), **7i** (Figure 3e) and **6e** (Figure 3(f)), the calculated values of *K*_SV_ were 0.129 µM^−1^ for **7j**, 0.069 µM^−1^ for **7i** and 0.035 µM^−1^ for **6e**. The reciprocal of these values (7.8 µM for **7j**, 14.5 µM for **7i** and 28.6 µM for **6e**) represents the inhibitor concentration leading to 50% quenching of CDC25B emission and possibly evaluates the *K*′_D_ of the enzyme•inhibitor complex.

**Figure 4.**
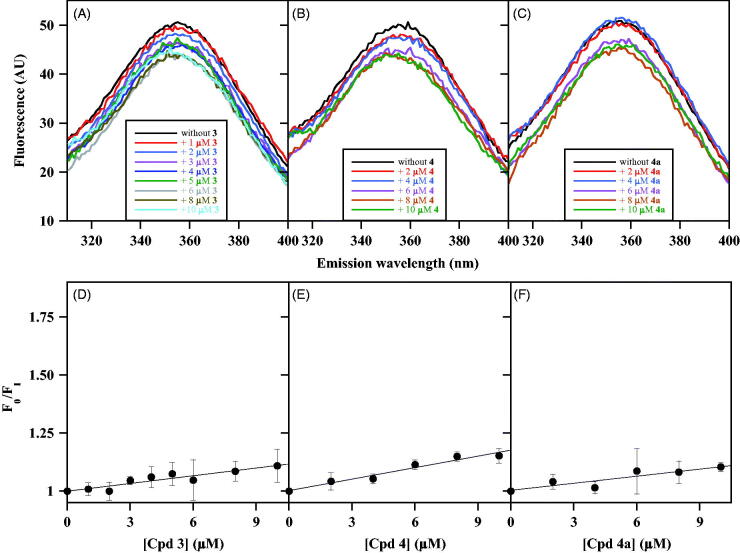
Effect of some uncompetitive inhibitors on the intrinsic fluorescence of recombinant CDC25B. The fluorescence spectra of 0.2 µM CDC25B in the absence or in the presence of the indicated concentrations of **3** (a), **4** (b) or **4a** (c) were recorded, normalised and corrected as indicated in Materials and Methods. An identical 1% (v/v) final concentration of DMSO is present in all samples. The data of fluorescence emission at 357 nm obtained in the absence (F_0_) or in the presence of each inhibitor concentration (F_I_) for **3** (d), **4** (e) or **4a** (f) were treated according to the Stern–Volmer equation.

Moving to the NPA derivatives approaching the uncompetitive inhibition, compounds selected from this class were **3**, **4** and **4a** with *K*_i_ values of 2.8 µM, 7.3 µM and 8.5 µM, respectively. The minimum effect produced by these putative uncompetitive inhibitors on the intrinsic fluorescence of CDC25B is shown in Figure 4. Indeed, differently from the results obtained with noncompetitive inhibitors, the emission spectra obtained in the presence of an increasing concentration of **3** (Figure 4(a)), **4** (Figure 4(b)) or **4a** (Figure 4(c)) exhibit a very low dose-dependent quenching. This behaviour also emerges from the low values of the straight-line slopes obtained when the data were analysed with the Stern-Volmer equation for **3** (Figure 4(d)), **4** (Figure 4(e)) or **4a** (Figure 4(f)). Indeed, the calculated values of *K*_SV_ (0.011 µM^−1^ for **3**, 0.016 µM^−1^ for **4**, and 0.010 µM^−1^ for **4a**) are significantly lower than those obtained with the noncompetitive inhibitors. As a consequence, the inhibitor concentration leading to 50% quenching of CDC25B emission is consistently greater than 50 µM for these inhibitors, a finding indicating that these compounds only display a very weak interaction with CDC25B, at least in the absence of OMFP.

### 3.4. Effect of the NPA derivatives on the intrinsic fluorescence of CDC25B-C473S in the absence or in the presence of OMFP

The weak binding interaction resulting from the intrinsic fluorescence experiments performed with the uncompetitive inhibitors **3**, **4** and **4a** suggests the hypothesis that these NPA derivatives are unable to form a stable CDC25B•inhibitor complex. Therefore, the possible formation of this complex in the presence of the substrate OMFP was investigated; in this case, one would expect that the minimum quenching of CDC25B emission observed with the uncompetitive inhibitor alone would increase when the enzyme is already bound to its substrate. To check this hypothesis, the mutant CDC25B-C473S turned to be useful, because this enzyme binds OMFP, but does not catalyse its hydrolysis.

**Figure 5.**
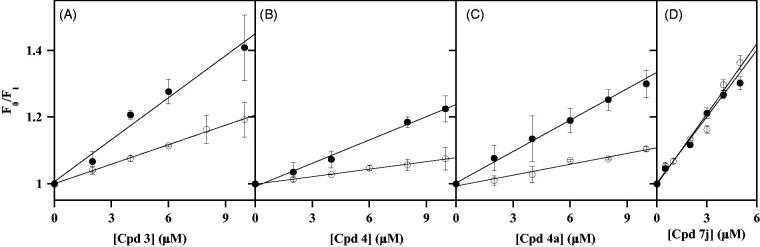
Effect of some inhibitors on the intrinsic fluorescence of recombinant CDC25B-C473S in the absence or in the presence of OMFP. The data of fluorescence emission at 357 nm used for drawing the Stern–Volmer plots were obtained from the fluorescence spectra reported in Figure S2, using the same protocol described in the legend to Figures 3 or 4. The F_0_/F_I_ ratios were calculated in the presence of the indicated concentrations of **3** (a), **4** (b), **4a** (c) or **7j** (d) either in the absence (open circles) or in the presence of 5 µM OMFP (filled circles).

The effects produced by the uncompetitive inhibitors **3**, **4** and **4a**, as well as by the noncompetitive inhibitor **7j**, on the intrinsic fluorescence of CDC25B-C473S in the absence or in the presence of 5 µM OMFP were investigated. In the absence of OMFP, the uncompetitive inhibitors cause a very low dose-dependent quenching of the CDC25B-C473S spectrum, similar to that exhibited by the wild-type CDC25B; on the other hand, in the presence of a saturating OMFP concentration, besides a roughly 50% reduction of fluorescence yield due to the quenching by the substrate, the inhibitors cause a consistent dose-dependent quenching (Supplementary Figure 2). Either in the absence or in the presence of OMFP no shift of the emission maximum is observed in the spectra and therefore, the data were analysed according to the Stern-Volmer equation for **3** (Figure 5(a)), **4** (Figure 5(b)) and **4a** (Figure 5(c)). In the absence of OMFP, low values of *K*_SV_ were obtained (0.019 µM^−1^, 0.008 µM^−1^ and 0.011 µM^−1^ for **3**, **4** and **4a**, respectively), similar to those measured with the wild-type CDC25B. This finding confirms that also the C473S mutant enzyme has a very weak interaction with these uncompetitive inhibitors. On the other hand, the slope of the straight lines substantially rises in the presence of OMFP and the measured values of *K*_SV_ increase to 0.042 µM^−1^, 0.023 µM^−1^ and 0.031 µM^−1^ for **3**, **4** and **4a**, respectively. The corresponding values of *K*′_D_ (24 µM, 43 µM and 32 µM for **3**, **4** and **4a**, respectively) indicate that OMFP strengthens the weak interaction of the enzyme with these uncompetitive inhibitors. Besides this relevant role of the substrate, the *K*′_D_ values of the uncompetitive inhibitors remain greater than those obtained for noncompetitive inhibitors, a finding probably reflecting the lower inhibition power exhibited by uncompetitive respect to noncompetitive inhibitors.

The effects on the intrinsic fluorescence of CDC25B-C473S in the absence or in the presence of 5 µM OMFP were probed even for a noncompetitive inhibitor, such as **7j** (Supplementary Figure 2). In this case, the dose-dependent quenching of the intrinsic fluorescence of CDC25B-C473S due to the inhibitor is evident without or with OMFP. Indeed, the straight lines of the corresponding Stern-Volmer plots obtained either in the absence or in the presence of OMFP are almost overlapping (Figure 5(d)). Therefore, these data confirm that the direct interaction between enzyme and inhibitor **7j** occurs in the absence of the substrate and it is not affected when the substrate binding pocket is occupied.

These overall effects on the intrinsic fluorescence of wild-type/mutant CDC25B caused by some NPA derivatives, acting as inhibitors of its phosphatase activity, reinforce the categorisation of these compounds in two groups according to a different inhibition mechanism. Indeed, **7j**, **7i** and **6e** directly interact with CDC25B, being capable to form the enzyme•inhibitor binary complex in the absence of the substrate; however, the formation of this complex is not affected when OMFP is bound to the enzyme, as shown for **7j**. Therefore, **7j**, **7i** and **6e** are endowed with an inhibition mechanism ranked as noncompetitive. On the other hand, for other inhibitors, such as **3**, **4** and **4a**, the presence of the substrate OMFP is required to strengthen an otherwise weak direct interaction between enzyme and inhibitor. In other words, **3**, **4** and **4a** may form a ternary complex, enzyme•OMFP•inhibitor, because the binding of the substrate is needed to permit a stable enzyme inhibitor interaction; therefore, these compounds approach the mechanism of inhibition ranked as uncompetitive.

The possible relationship between binding interaction and inhibition power of the selected NPA noncompetitive and uncompetitive inhibitors of CDC25B has been evaluated (Table 1). The parameters used for the comparison are the *K*′_D_ values obtained from fluorescence studies reported in this work and the corresponding *K*_i_ previously obtained in kinetic studies of CDC25B phosphatase inhibition^18^. The binary complex formed by CDC25B with the typical noncompetitive inhibitors **7j**, **7i** or **6e**, has a consistently greater *K*′_D_ compared to *K*_i_. Besides this difference, possibly due to the different experimental conditions used for measuring *K*′_D_ or *K*_i_, it is important to note that both parameters increase in the same order (**7j** < **7i** < **6e**), as confirmed by the *K*′_D_/*K*_i_ ratio of 9.7, 10.4 and 10.6 for **7j**, **7i** and **6e**, respectively. Therefore, the decreased inhibition power of **7i** or **6e** compared to **7j** probably reflects their lower binding interaction with CDC25B. Moving to the uncompetitive inhibitors **3**, **4** and **4a**, the *K*′_D_ values considered for the comparison are those obtained for the complex formed by CDC25B-C473S in the presence of the substrate OMFP. Also in this case the *K*′_D_ are consistently greater than the corresponding *K*_i_ values, as also emerging from the *K*′_D_/*K*_i_ ratio of 8.6, 5.9 and 3.8 for **3**, **4** and **4a**, respectively. However, in spite of the different values of the ratio, the significantly weaker inhibition power of **4** and **4a** with respect to **3** may be explained with their lower binding interaction with the enzyme. All these data suggest that the different inhibition power and mechanism exhibited by the NPA derivatives of NSC28620 may be explained by a modulation of their direct or substrate-mediated interaction with the enzyme, as a consequence of the structural changes operated on the lead compound.

**Table 1.** Binding interaction of selected NPA derivatives of NSC28620 towards CDC25B and CDC25B-C473S and relative inhibition mechanism

	*K′*_D_ of the complex (µM)^a^				
Inhibitor	Free CDC25B•inhibitor	Free CDC25B-C473S•inhibitor	OMFP-bound CDC25B-C473S•inhibitor	*K*_i_ (µM)^b^	Inhibition mechanism
**7j**	7.8	14.3	15.1	0.8	noncompetitive
**7i**	14.5	n.d.	n.d.	1.4	noncompetitive
**6e**	28.6	n.d.	n.d.	2.7	noncompetitive
**3**	≫50	>50	24	2.8	uncompetitive
**4**	>50	≫50	43	7.3	uncompetitive
**4a**	≫50	>50	32	8.5	uncompetitive

^a^ Values are the reciprocal of the *K*_SV_ obtained from the intrinsic fluorescence studies reported in this work (Stern–Volmer plots in Figures 3–5).

^b^ Values from the kinetic studies of CDC25B phosphatase inhibition^18^.

n.d.: not determined.

Our fluorescence studies represent a powerful tool to shed light on the inhibition mechanism exerted by small molecules on a target enzyme. Indeed, the combination of kinetic studies of CDC25B phosphatase inhibition by NPA derivatives and fluorimetric investigation on their binding interaction with the enzyme led to a detailed understanding of the inhibition mechanism of these small molecules based on a direct (noncompetitive) or substrate-mediated (uncompetitive) interaction with the target enzyme. Furthermore, the finding that three members of the same group of inhibitors keep a fluorimetric behaviour specific for the type of inhibition might point to the statistical relevance of this investigation, thus allowing an enlargement of the assignment (noncompetitive or uncompetitive inhibition) to the other NPA derivatives.

### 3.5. Pro-apoptotic effect exhibited by the NPA derivative 4a in melanoma cell lines

**Figure 6.**
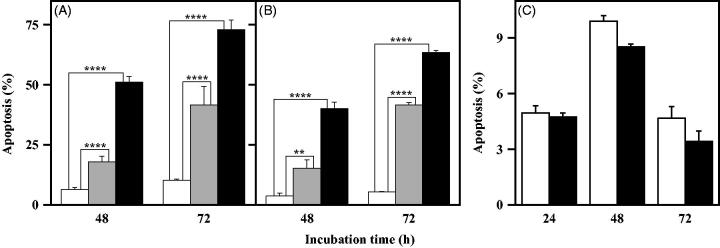
Apoptotic effect of **4a** in A2058, A375 and BJ-5ta cell lines. A2058 (a), A375 (b) and BJ-5ta (c) cells were treated with 0.5% (v/v) DMSO (white bars), 5 μM **4a** (grey bars) or 10 μM **4a** (black bars), and apoptosis was evaluated after the indicated incubation time through the determination of the number of cells with a subdiploid DNA content. Data from triplicate experiments were expressed as a percentage and reported as the mean ± SE. **, *p* < 0.01; ****, *p* < 0.0001 compared to untreated cells.

Once assessed the inhibition mechanism exerted by the NPA derivatives on purified CDC25B, we have made a deeper insight on their possible mechanism of action in a cellular context, such as melanoma, one of the most aggressive and therapy-resistant human cancers. Under this concern, the previous study on the biological effects exerted by all NPK and NPA derivatives in A2058 and A375 melanoma cell lines^18^ revealed that only one of these compounds, **4a**, exerted a toxic effect in melanoma cells, when added at a relatively low dose. In particular, this compound reduced the cell growth rate, provoked an arrest of cells in G2/M phase of cell cycle and reduced the protein levels of pCDK1. This behaviour could be indicative of a DNA damage induced by **4a**. It is noted that the entry in each phase of cell cycle is strictly supervised by cell-cycle checkpoint systems that are responsible for the arrest of cell cycle, as a response to DNA damage. A block in G2/M phase prevents the entry into mitosis, thus allowing the onset of a number of processes devoted to repair the DNA damage. The activation of CDC25 phosphatases, in particular CDC25C, is crucial for the progression through G2/M checkpoint^36^. Hence, the inhibition of CDC25 can contribute to the arrest of cell cycle in G2/M phase; however, if the arrest is prolonged and the DNA damage is not repaired, cells can activate an apoptotic programme in order to avoid the transfer of DNA mutated to daughter cells^37^. Therefore, the possible activation of an apoptotic programme during treatment of melanoma cells with **4a** was evaluated. To this aim, A2058 and A375 cells were treated with 5 or 10 µM **4a** at different times and apoptosis was monitored cytofluorimetrically after PI incorporation (Figure 6). A dose- and time-dependent increase of cells with a hypodiploid DNA content is evident in both A2058 (Figure 6(a)) and A375 cells (Figure 6(b)). In particular, the increase of apoptotic cells, already significant after 48-h treatment in the presence of either 5 or 10 µM **4a**, becomes more pronounced after 72-h treatment in both melanoma cells. The effect of 10 µM **4a** was also tested in the nontumorigenic fibroblast cell line BJ-5ta. As shown in Figure 6(c), **4a** does not exert any apoptotic effect in these nonmalignant cells after 24-, 48- or 72-h treatment. Therefore, the toxic effect exerted by **4a** in melanoma cells seems to be specific, because a nontumour cell line, such as BJ-5ta, is not responsive to this compound.

**Figure 7.**
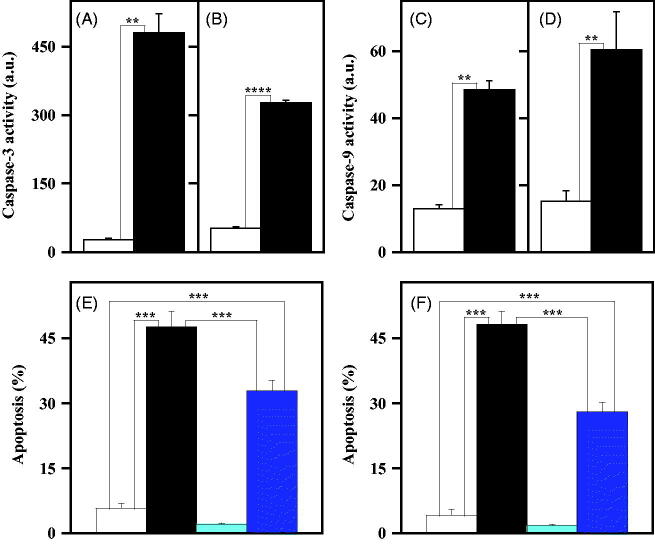
Involvement of caspase-3 and -9 in the apoptotic programme induced by **4a** in melanoma cells. (a, b, c, d) Effect of **4a** on the caspase-3 and caspase-9 activity in melanoma cells. A2058 (a, c) and A375 (b, d) cells were incubated for 24 h with 0.5% (v/v) DMSO (white bars) or 10 μM **4a** (black bars). The enzymatic activity of caspase-3 (a, b) and caspase-9 (c, d) was reported as arbitrary units (a.u.). Data from triplicate experiments were reported as the mean ± SE. **, *p* < 0.01; ****, *p* < 0.0001 compared to untreated cells. (e, f) Effect of Z-VAD-FMK, a pan-caspase inhibitor, on the apoptosis induced by **4a** in melanoma cells. A2058 (e) and A375 (f) cells were incubated for 48 h with 0.5% (v/v) DMSO (white bars), 10 μM **4a** (black bars), 100 µM Z-VAD-FMK (cyan bars) or 10 μM **4a** plus 100 µM Z-VAD-FMK (blue bars). The apoptosis was evaluated through the determination of the number of cells with a subdiploid DNA content. Data from triplicate experiments were expressed as a percentage and reported as the mean ± SE. ***, *p* < 0.001 compared to respective control cells.

The pro-apoptotic potential of **4a** in melanoma cells was further investigated by measuring the enzymatic activity of caspase-3 and -9, representing the final effector of apoptosis, and one of the main mediators of the intrinsic apoptotic programme, respectively^38^. As shown in Figure 7, the treatment of melanoma cells with 10 µM **4a** induced a statistically significant increase of the enzymatic activity of caspase-3 (Figure 7(a,b)) and caspase-9 (Figure 7(c,d)), an enhancement clearly evident for both A2058 (Figure 7(a,c)) and A375 (Figure 7(b,d)). To verify whether the apoptosis induced by **4a** in melanoma cells was caspase-mediated, the cytofluorimetric analysis of apoptosis was also conducted in the presence of an irreversible pan-caspase inhibitor, such as Z-VAD-FMK. Melanoma cell lines were treated for 48 h with **4a** in the presence or in the absence of Z-VAD-FMK; a partial but significant reduction of the apoptosis level was observed in both A2058 (Figure 7(e)) and A375 (Figure 7(f)) cells upon exposure to the caspase inhibitor.

**Figure 8.**
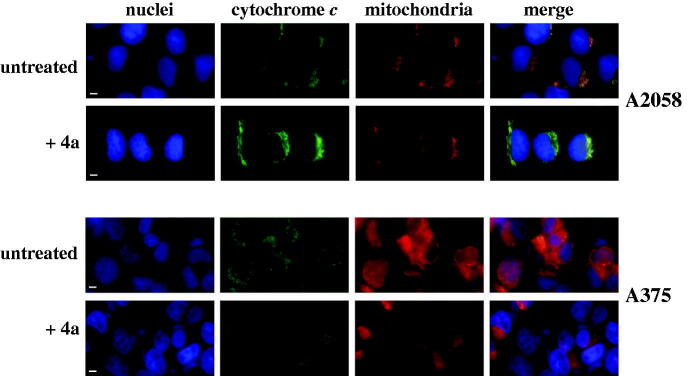
Immunofluorescence analysis of cytochrome *c* subcellular localisation in melanoma cells. A2058 and A375 cells were untreated (0.5% v/v, DMSO) or treated with 10 μM **4a** for 24 h. The cell nuclei were labelled with DAPI (blue channel); the cytochrome *c* immunofluorescent staining (green channel) was performed using a polyclonal anti-Cyt *c* antibody, followed by FITC-conjugated antibody; mitochondria were stained with MitoTracker Red (red channel). The degree of spatial overlap in cytochrome *c* immunostaining and MitoTracker Red staining is shown by yellow fluorescence, corresponding to merge of red and green channel. Scale bar, 10 µm; magnification × 40.

**Figure 9.**
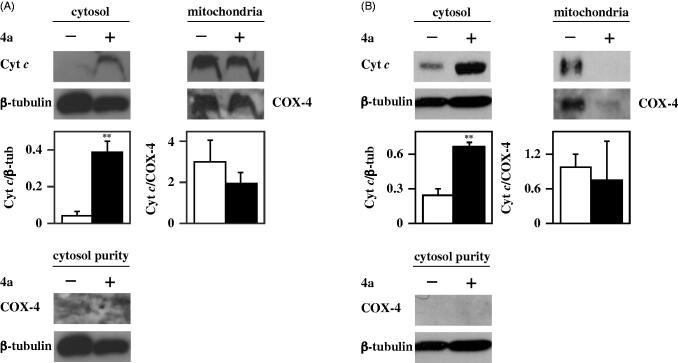
Effect of **4a** on cytochrome *c* subcellular localisation in melanoma cells. A2058 (a) and A375 (b) cells were incubated for 24 h with 0.5% (v/v) DMSO (white bars) or 10 μM **4a** (black bars), and then the cytosolic and mitochondrial protein extracts were prepared for Western blotting analysis, using β-tubulin or COX-4 as loading controls of the cytosolic or mitochondrial fraction, respectively. Purity of the cytosolic fraction was monitored by using β-tubulin or COX-4 as specific cytosolic or mitochondrial marker, respectively. The densitometric evaluation of three independent experiments was reported as the mean ± SE. **, *p* < 0.01 compared to untreated cells.

The increase of caspase-9 activity suggests that the apoptotic programme induced by **4a** in melanoma cells could be mitochondria-mediated. It is noted that cytochrome *c*, a small protein normally localised in the mitochondria, moves into the cytosol upon some death stimuli, thus leading to the activation of caspase-9^39^. Therefore, an investigation on the cytosolic translocation of cytochrome *c*, the main regulator of the intrinsic apoptotic pathway, was carried out through immunofluorescence and Western blotting experiments. In particular, A2058 and A375 cells, untreated or treated with 10 µM **4a**, were subjected to immunofluorescence labelling with an anti-cytochrome *c* antibody (Figure 8). In untreated cells, cytochrome *c* (green signal) is essentially localised in the mitochondria (red signal), as confirmed by the yellow merged signal. On the other hand, treatment with **4a** induces a cytosolic translocation of cytochrome *c*, as clearly indicated by the appearance of green fluorescence in the cytosol. These results were confirmed by Western blotting experiments using fractioned protein extracts (cytosol and mitochondria) of melanoma cells incubated with **4a**. Indeed, this compound induces a significant cytosolic translocation of cytochrome *c* in both A2058 (Figure 9(a)) and A375 (Figure 9(b)) cells. All these data indicate that the cell cycle arrest in G2/M provoked by **4a** in melanoma cells results in the activation of an apoptotic programme, mainly mitochondria- and caspase-mediated.

### 3.6. Effect of 4a on typical markers of cell cycle progression, apoptosis and proliferation

**Figure 10.**
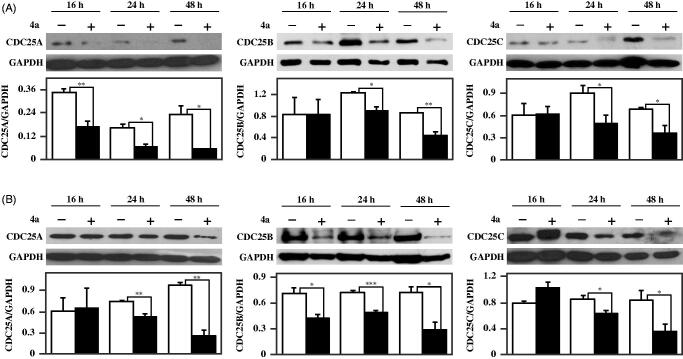
Effect of **4a** on CDC25A, -B and -C protein levels in melanoma cells. A2058 (a) and A375 (b) cells were incubated with 0.5% (v/v) DMSO (white bars) or 10 μM **4a** (black bars) for the indicated incubation time. Western blotting analysis was performed on total cellular extracts, using GAPDH as loading control. The densitometric evaluation of three independent experiments was reported as the mean ± SE. *, *p* < 0.05; **, *p* < 0.01; ***, *p* < 0.001 compared to untreated cells.

The response of melanoma cells to **4a** prompted an investigation on how this inhibitor affects the levels of typical protein markers of cell cycle progression, apoptosis and proliferation. In a previous work we have reported that **4a** arrested the cell cycle of melanoma cells in G2/M phase, and provokes an increase of protein levels of pCDK1, the inactive form of CDK1^18^. It is known that the activation of CDK1 through the dephosphorylation of its Tyr15 catalysed by CDC25 promotes the cell entry into mitosis; therefore, the inhibition of CDC25, retaining CDK1 in an inactive state, impedes the cell cycle progression and can contribute to cell death. Furthermore, CDC25 exerts an anti-apoptotic role, by suppressing some apoptotic pathways either directly or indirectly. Indeed, CDC25 inhibits Apoptosis Signal-regulating Kinase 1 (ASK1) through dephosphorylation of Thr838 or inhibits apoptosis through activation of CDK1, that in turn phosphorylates and inactivates caspase-9^40–42^. Hence, the effects induced by **4a** in melanoma cells could be related to its inhibitory capacity towards CDC25 activity also in a cellular system. To this aim, we have also evaluated the protein levels of CDC25A, –B and –C during treatment of melanoma cells with **4a**. As shown in Figure 10, **4a** provokes a reduction of protein levels of all three forms of CDC25 enzymes in both A2058 (Figure 10(a)) and A375 (Figure 10(b)) melanoma cells, essentially after 24- and 48-h of incubation. Hence, we can infer that the inhibition of CDC25 enzymatic activity and the reduction of CDC25 protein levels concur to mediate the toxic effect induced by **4a** in melanoma cells.

**Figure 11.**
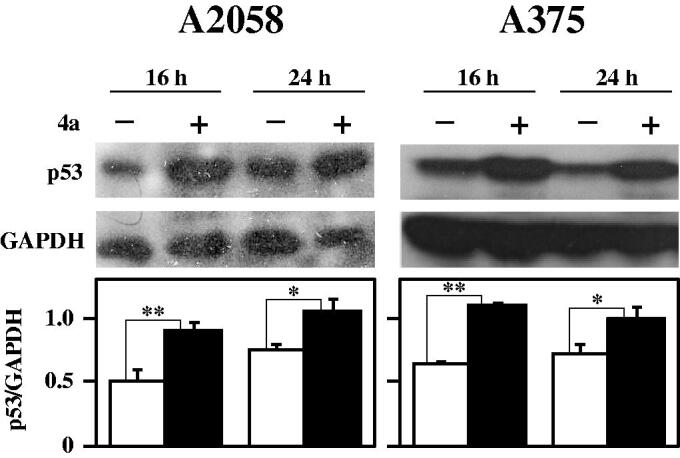
Effect of **4a** on p53 protein levels in melanoma cells. A2058 and A375 cells were incubated with 0.5% (v/v) DMSO (white bars) or 10 μM **4a** (black bars) for the indicated incubation time. Western blotting analysis was performed on total cellular extracts, using GAPDH as loading control. The densitometric evaluation of three independent experiments was reported as the mean ± SE. *, *p* < 0.05; **, *p* < 0.01 compared to untreated cells.

The integrity of cell genome is under a strict control by a number of proteins devoted to cell cycle regulation and apoptosis. Among them, p53, named the “genome guardian”, acts as tumour suppressor protein, performing a pivotal role in this control. This protein, mediating the activation of key mechanisms involved in the eradication of damaged cells, exerts a fundamental role in the processes of neoplastic transformation^43^^,^^44^. In addition, p53 is required for a good response to many forms of tumour therapies and thus is an appealing target for anticancer drug studies^45^. As shown in Figure 11, a clear and significant increase of p53 is evident in A2058 and A375 melanoma cells after 16- and 24-h treatment with **4a**. This finding is in agreement with the negative role exerted by p53 in the gene expression of both CDC25B and –C^46^^,^^47^ and with the previous described results on CDC25 down-regulation.

**Figure 12.**
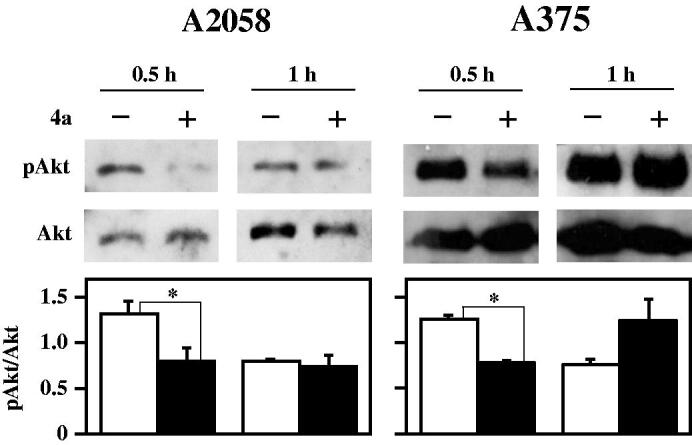
Effect of **4a** on pAkt protein levels in melanoma cells. A2058 and A375 cells were incubated with 0.5% (v/v) DMSO (white bars) or 10 μM **4a** (black bars) for the indicated incubation time. Western blotting analysis was performed on total cellular extracts, using Akt as loading control. The densitometric evaluation of three independent experiments was reported as the mean ± SE. *, *p* < 0.05 compared to untreated cells.

A high percentage of melanomas harbours a mutation in BRAF, a gene encoding a member of the mitogen-activated protein kinase (MAPK) signalling pathway, that modulates cell growth, proliferation and survival^48^^,^^49^. Treatment of melanoma patients with B-Raf inhibitors, such as vemurafenib, is characterised by an initial phase where a strong reduction of tumour is obtained; unfortunately, within a few months, many patients loose the durable response to B-Raf inhibitors because of the occurrence of drug resistance^50–52^. This problem can be due to a compensatory activation of other cell surviving pathways, bypassing the anti-proliferative effects of B-Raf inhibitors^53^. Indeed, the phosphatidylinositol 3-kinase/protein kinase B (PI3K/AKT) pathway is frequently activated in primary melanomas^52^ and the use of B-Raf inhibitors paradoxically activates the AKT signalling pathway, that promotes the tumour survival of BRAF-mutant melanoma cells^54^^,^^55^^,^. Furthermore, in tumour cells, the UV exposure can activate the PI3K/AKT‐dependent mechanism that stabilises CDC25A and induces its translocation into the cytoplasm, where it does not affect the cell cycle progression, but acts as anti-apoptotic factor^56^. All these observations prompted an evaluation on the pAkt protein levels during treatment of melanoma cells with **4a**. Data in Figure 12 show a significant and early decrease of pAkt after 30-min treatment with **4a**. This finding is very interesting, because the combined usage of **4a** and conventional B-Raf inhibitors could contribute to reduce the development of drug resistance in melanomas. Under this concern, the reduction of pAkt protein levels is evident in the primary melanoma cell line A375, which is sensitive to vemurafenib, as well as in the metastatic and vemurafenib-resistant cell line A2058.

In the complex, this molecular analysis on the effects of **4a** in melanoma cells suggests that the toxic effect of this compound might involve both the inhibition of the CDC25 activity/protein levels and the modulation of pivotal cellular surviving pathways.

## 4. Conclusions

Among CDC25 inhibitors, compounds with the NPA scaffold are particularly attracting for the design of molecules endowed with potential anticancer properties, because their nonquinonoid structure reduces the unspecific side toxic effects usually observed with the most known quinonoid CDC25 inhibitors. Hence, the detailed biochemical characterisation of the inhibition mechanism possessed by NPA derivatives described in this work and the molecular processes involved in the biological effects observed with a highly cytotoxic member of these molecules improve the basic knowledge on CDC25 inhibitors and represent the starting point for the discovery and production of more potent bioactive molecules. Indeed, the drug discovery studies are mainly focussed on the obtainment of molecules characterised by a high specificity against selected cancer cells, minimum undesired side toxic effects and reduced appearance of drug resistance. Under this concern, the specific responsiveness of melanoma cells, a highly resistant cancer type, to the bioactive compound **4a** is particularly interesting, because this molecule affects alternative survival pathways responsible for the acquirement of drug resistance usually observed during the therapeutic treatment of melanoma with conventional agents. These observations could allow the development of anticancer strategies based on the cotreatment of melanoma with different drugs cotargeting independent survival pathways or the combination of multitargeted therapy and immunotherapy.

## Supplementary Material

Supplemental Material
