# Supplementary material for: Inhibition mechanism of naphthylphenylamine derivatives acting on the CDC25B dual phosphatase and analysis of the molecular processes involved in the high cytotoxicity exerted by one selected derivative in melanoma cells
Source: J Enzyme Inhib Med Chem. 2020 Sep 29;35(1):1866–78. doi: 10.1080/14756366.2020.1819257 (PMC7580834; doi:10.1080/14756366.2020.1819257)
Supplement: Supplemental Material [file IENZ_A_1819257_SM6238.pdf]

Supplemental Material for

Naphthylphenylamine derivatives acting on the CDC25B dual phosphatase: An insight on the inhibition mechanism by fluorimetric analysis and molecular processes involved in the high cytotoxicity of a selected derivative in melanoma cells

Federica Aliotta,[†,¶] Rosarita Nasso,[#,¶] Rosario Rullo,[§] Alessandro Arcucci,[˩] Angelica Avagliano,[˩] Martina Simonetti[†], Gennaro Sanità[†], Mariorosario Masullo,[#] Antonio Lavecchia,[⌘] Maria Rosaria Ruocco,[†,*] and Emmanuele De Vendittis[†,*]

[†] Department of Molecular Medicine and Medical Biotechnology, University of Naples Federico II, Via S. Pansini 5, 80131 Naples, Italy

[#] Department of Movement Sciences and Wellness, University of Naples "Parthenope", Via F. Acton 38, 80133 Naples, Italy

[§] Institute for the Animal Production Systems in the Mediterranean Environment, Via Argine 1085, 80147 Naples, Italy

[˩] Department of Public Health, University of Naples Federico II, Via S. Pansini 5, 80131 Naples, Italy

[⌘]Department of Pharmacy, "Drug Discovery" Laboratory, University of Naples Federico II, Via D. Montesano, 49, 80131 Naples, Italy

Supplemental Figures

Figure S1. Intrinsic fluorescence of recombinant CDC25B and CDC25B-C473S. The fluorescence spectra of 0.2 µM CDC25B (black line) or 0.2 µM CDC25B-C473S (red line) were recorded as indicated in Materials and Methods.

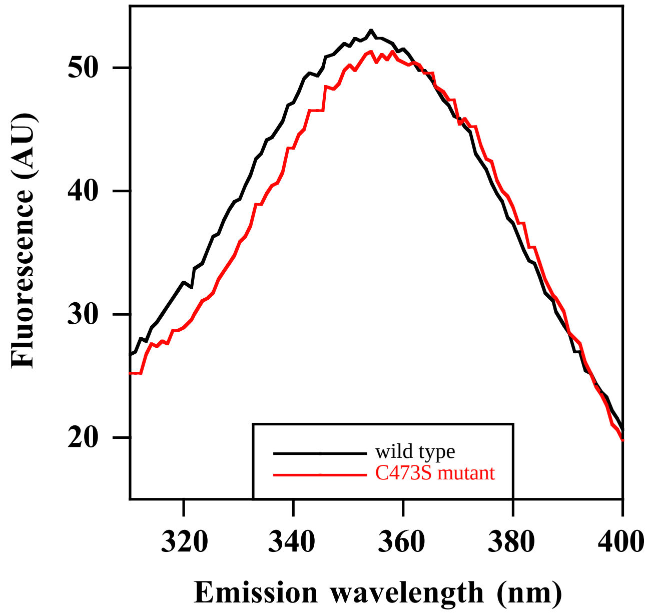

Figure S2. **Effect of the un-competitive inhibitors 3, 4 and 4a or the non-competitive inhibitor 7j on the intrinsic fluorescence of recombinant CDC25B-C473S in the absence or in the presence of OMFP**. The fluorescence spectra of 0.2 µM CDC25B-C473S were recorded in the absence (A,C,E,G) or in the presence of 5 µM OMFP (B,D,F,H) without or with the indicated concentrations of Cpd **3** (A,B), Cpd **4** (C,D), Cpd **4a** (E,F) or Cpd **7j** (G,H). All spectra were normalized and corrected as indicated in Materials and Methods. Each experimental point contained an identical final concentration of DMSO (1%, v/v) and methanol (0.25%, v/v). Compared to those recorded in the absence of OMFP, spectra in the presence of the substrate showed a roughly 50% reduction of fluorescence emission due to the quenching caused by 5 µM OMFP.

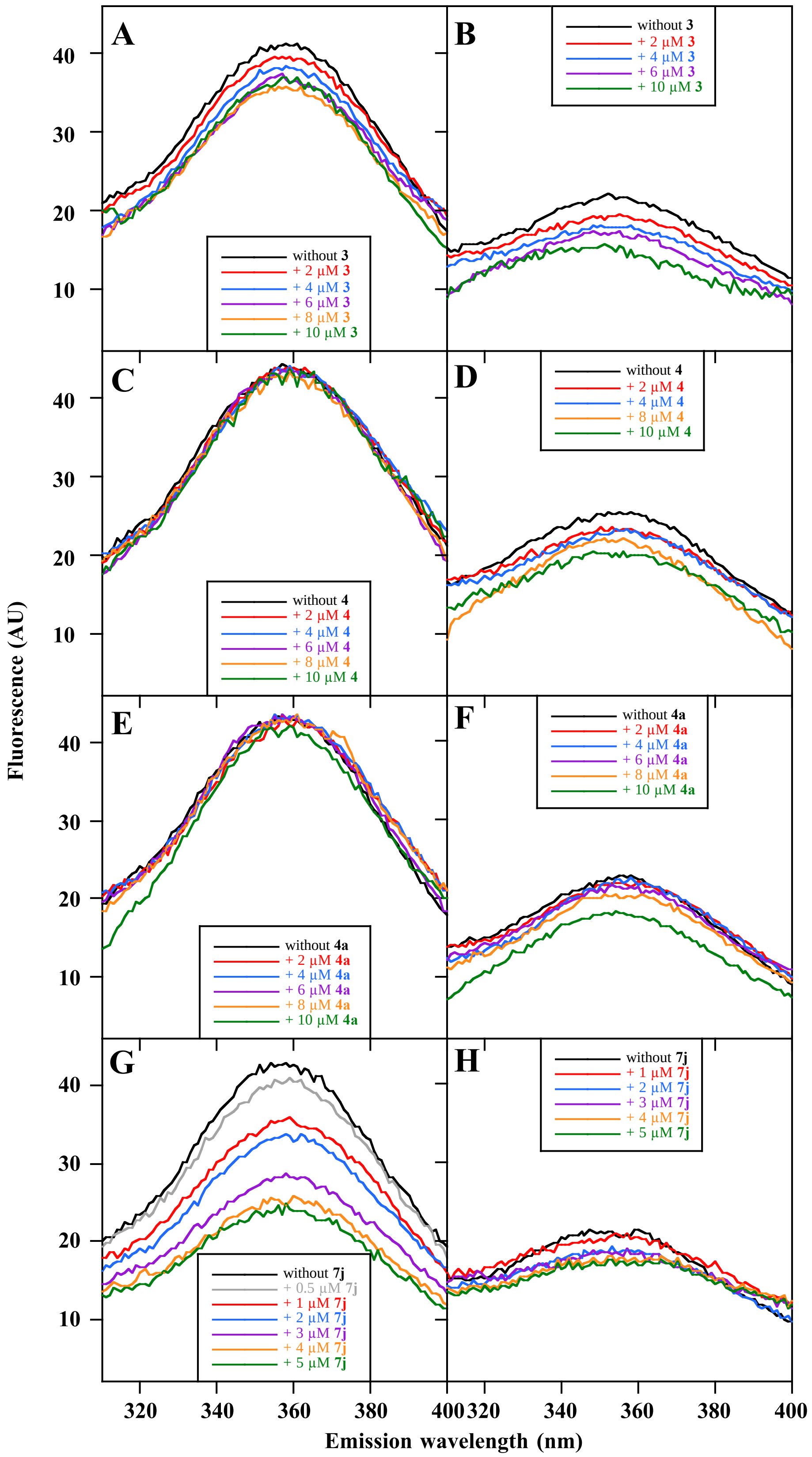

A
B
C
D
E
F
G
H
without 3
+ 2 μM 3
+ 4 μM 3
+ 6 μM 3
+ 8 μM 3
+ 10 μM 3
without 4
+ 2 μM 4
+ 4 μM 4
+ 6 μM 4
+ 8 μM 4
+ 10 μM 4
without 4a
+ 2 μM 4a
+ 4 μM 4a
+ 6 μM 4a
+ 8 μM 4a
+ 10 μM 4a
without 7j
+ 0.5 μM 7j
+ 1 μM 7j
+ 2 μM 7j
+ 3 μM 7j
+ 4 μM 7j
+ 5 μM 7j
Fluorescence (AU)
Emission wavelength (nm)
